# Transfer of Health for All policy – What, how and in which direction? A two-case study

**DOI:** 10.1186/1478-4505-2-8

**Published:** 2004-12-07

**Authors:** Leena Tervonen-Gonçalves, Juhani Lehto

**Affiliations:** 1 Tampere School of Public Health, 33014 University of Tampere, Finland

## Abstract

**Background:**

This article explores the transfer of World Health Organization's (WHO) policy initiative *Health for All by the Year 2000 *(HFA2000) into national contexts by using the changes in the public health policies of Finland and Portugal from the 1970's onward and the relationship of these changes to WHO policy development as test cases. Finland and Portugal were chosen to be compared as they represent different welfare state types and as the paradigmatic transition from the old to new public health is assumed to be related to the wider welfare state development.

**Methods:**

The policy transfer approach is used as a conceptual tool to analyze the possible policy changes related to the adaptation of HFA into the national context. To be able to analyze not only the content but also the contextual conditions of policy transfer Kingdon's analytical framework of policy analysis is applied.

**Conclusions:**

Our analysis suggests that no significant change of health promotion policy resulted from the launch of HFA program neither in Finland nor in Portugal. Instead the changes that occurred in both countries were of incremental nature, in accordance with the earlier policy choices, and the adaptation of HFA program was mainly applied to the areas where there were national traditions.

## Introduction

The World Health Organization (WHO) launched a policy framework called Health for All by the Year 2000 (HFA2000), in 1978, and has since then been advocating this framework for health policy making to all its member states [1,2]. This paper explores the transfer of HFA policy into national contexts by using the changes in public health policy of Finland and Portugal from the 1970's onward and their relationship to WHO policy development as test cases. Finland and Portugal were chosen to be the cases observed as they represent different welfare state types and as the paradigmatic shift from the old to new public health is assumed to be related to the wider welfare state development. The development of the welfare state constitutes the frame of reference for the analysis of transfer of HFA policy.

Policy transfer is a theoretical perspective that has been used to describe the spread of policy ideas from one political setting to another [3]. Most studies have concentrated on studying the transfer between countries, here the transfer is assumed to be mediated through an international organization (WHO) to its member states. Our aim is to locate the transfer of HFA policy in a broader conceptual framework. This entails clarifying the theoretical and political assumptions inherent in HFA policy as well as studying the transfer process in the historical context of broader welfare state development.

In order to analyze the transfer of HFA policy it is necessary to recognize that HFA policy is not a totally coherent health strategy that can be defined in one compact and consensual manner. The ambiguous nature of HFA policy stems from fact that it is constructed in various policy documents drawn up in temporally and contextually different situations. We refer to the following four central documents and the ideal model of public health policy they construct when we speak of HFA policy: the Declaration of Alma-Ata (1978) [1], Targets in support of the European regional HFA strategy (1985) [4], the Ottawa Charter (1986) [5], Health21 for Europe (1999) [6]. There are points of convergence in the picture of the ideal policy model these documents transfer, but also differences linked to the evolution of temporal macro-political cycles (collapse of colonialism, new international economic order, expansion of the welfare state, collapse of communist regimes in Eastern Europe, globalization and the crisis of the welfare state) or to the regional characteristics (European vs. global). Thus when we speak of HFA we refer to the HFA policy constructed in the aforementioned documents. To be able to analyze the adaptation of HFA in national contexts we have concentrated on examining three aspects of it: primary health care, community approach and healthy public policies. Based on the analysis of these aspects the study aims to explore how since the 1970's a number of essential aspects of health promotion policy have changed in Finland and in Portugal in relation to the ideas of HFA.

While a few studies have addressed the spreading of the HFA policy to the member states [7-11], this is quite seldom based on any theory of policy change or policy transfer. Also, while most of these studies have either been descriptive in nature or focused on evaluating national policies in the program level by verifying program's outcomes or situational validity of its objectives, we aim to analyse the policies in a broader societal context by taking into account the societal-level vindication as well as the political context of health policies [12].

In the policy transfer literature past policies, present policy complexity and the question of policy feasibility are seen as possible policy constraints. Likewise factors such as identical past policies or similar ideology can be seen to facilitate the transfer [3]. Locating the transfer of HFA policy in the context of existing public health policies and the wider political and social contexts of the countries in question offers one means to identify essential capacities, constraints and conditions for the adaptation of this particular policy innovation.

To be able to analyze not only the content (*What was transferred?*) but also the contextual conditions (*How/why did this happen?*) of policy transfer we use Kingdon's (1986) analytical framework of policy analysis [13]. According to Kingdon, a policy change process is conditioned by three analytically distinct streams: problem, policy and politics stream. Problem stream brings issues to the political agenda, while policy stream, which consists of experts, produces solutions and alternatives to policy problems. From these alternatives the politics stream then determines what, if any, are politically feasible alternatives to be adapted. A window of opportunity is open for a major policy change only if these three different streams of policy making process coexist simultaneously.

Policy transfer may occur at different stages of the policy making process. In this paper we will focus mainly on the agenda-setting and policy formulation phases. These phases can be regarded as a valuable starting point for the further development and implementation of HFA at the national level. The policy transfer approach is used as a conceptual tool to analyze the possible policy changes that the adaptation of HFA into the national context may have caused.

## Method and materials (see figure 1)

We aim to identify concrete examples of transfer related changes in the content of formal government documents such as laws, reports, strategies and government programs. The detailed analysis of these documents provides some evidence of policy transfer. Non-formal government documents, evaluative reports, studies and relevant discussion are also used as material. The analysis of the policy documents was supported by expert interviews conducted in both of the countries in 2003–2004 for the purposes of this study. Historical reading of the documents can provide evidence about the time frame of policy change. In Portugal the first health strategy was published in 1999 [14], and thus the primary material for the analysis of governmental health policy before 1999 is government programs [15]. Finnish health promotion policy and its relevant documents [16-18] have been evaluated twice by an international review group [19,20] and several times by Finnish public health experts and national committees [20] and thus the analysis of the Finnish case is rather based on these evaluations and reviews than on the programs.

**Figure 1:**
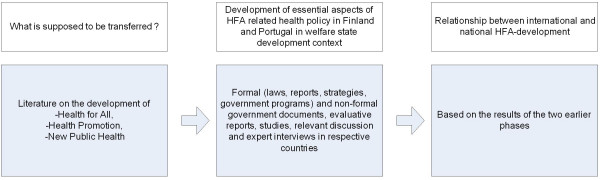
The Analytical Framework of the Study

## Results

### HFA as a rethinking of public health policy

WHO advocated "Health for All" as a rethinking of and challenge for reform in the national public health policies of the member states. HFA was frequently understood as a policy for developing countries focusing on advocacy for linking public health aims with broad social and environmental development policy at the local level, instead of investing in hospital medicine for the elites of the country. The core idea of the Alma-Ata Declaration (1978) was to advocate such a policy under the concept of *primary health care*.

In most OECD countries there already was some organizational form of primary *medical *care. Many health policy makers thought that HFA does not apply to OECD countries. Others argued that the idea of a comprehensive social and intersectoral health policy under the banner of primary health care also challenged the OECD countries. According to this understanding, Europe, too, was to develop its own HFA policy [21].

Seven years after Alma-Ata, in 1985, the WHO Regional Committee for Europe adopted its own HFA policy. It advocated a comprehensive, intersectoral and participatory health policy aiming at health gain and equity in health [4]. The conceptual differences between the Global and the European HFA are significant. The European HFA located primary health care as one aspect of "appropriate care" and to the basic level in the organization of health services. Both the Global and the European HFA argued for a comprehensive and intersectoral health policy. However, the meaning of these concepts is dependent on the context in which they are used. It may be argued that in the European HFA, the context is a welfare state – at that time either state capitalistic or state socialistic – with its numerous institutions and administrative sectors. The context in the Global HFA was a general and broad social and economic development of so-called developing countries.

The concept of HFA was accompanied by the introduction of two other challenging concepts: health promotion [5] and new public health [22]. All three were rhetorically contrasted to something that was called old or dominant way of thinking and making health policy that was characterized as focusing on hospital and cure, following a biomedical model and applying a narrow understanding of health and determinants of health.

Each of these three concepts had their own history and points of reference. For example, health promotion was mainly developed from a critical assessment of the health education of the 1970's [23]. New public health was advocated as a response to the change in the disease panorama, which meant that instead of hygiene, physical environment and vaccinations the new focus of interventions was to be on the social, cultural and political determinants of lifestyles and health [24]. HFA advocated an outcome-oriented health policy implemented by a wide range of social and economic institutions instead of focusing on the supply of medical care inputs.

The Ottawa Charter on Health Promotion mentions peace, shelter, education, food, income, a stable ecosystem, sustainable resources, social justice and equity as basic prerequisites for health [5]. In addition to advocating reorientation in health services and the development of personal health skills, the Charter also includes in health promotion foci a wide range of public policies, communities and daily social and physical environments. The WHO Vienna Dialogue (1986) even concluded that the best health promotion policy is a good social policy [25]. Taking into consideration that OECD had declared, in 1979 [26], the "Crisis of the Welfare State", we may locate the conceptual innovation of health promotion in OECD countries as advocacy for certain aspect of welfare state reform, as one remedy for the "crisis".

The European version of HFA may also be read from a welfare state reform advocacy perspective. The European HFA strongly advocates a broad health policy managed by objectives [21]. The strong management by objectives advocacy in the European HFA [4,6] links it with the managerialistic reform agenda of the welfare state [e.g. [27,28]]. Managerialism seems to be less prominent in the Ottawa Charter. Rather, it is possible to claim that the Charter has been influenced by ideas to develop welfare states leaning on social and community movements [29].

For the purposes of this study, we may conclude that there are three related concepts, health for all, health promotion and new public health that have a lot in common in their critique of the hospital focused and biomedically oriented health policy paradigms. They all advocate a broader socio-political orientation for health policy. However, they do not have a common idea of what this broader orientation is. We find it helpful to distinguish at least three different orientations. The first is the broad social and economic development context where the Alma-Ata Declaration located the radical local development program under the concept of primary health care. The second is the managerialistic welfare state reform context where the European HFA located the proposed health policy guided by HFA targets. A third orientation has been developed under the banner of the Ottawa Charter. This third variant may also be located in a welfare state reform context, but in a different idea of reform emphasizing community development.

### Finland and the HFA challenge

Finland experienced an extremely rapid urbanization phase in the 1960's and the 1970's. A large part of the population moved from the rural areas to industrial and service workplaces in the urban centers. The proportion of working people earning their main income from agriculture decreased from 26% to 10% in 1950–1980 [30]. A significant part – about 7 % of the population – even moved outside the country, to Sweden. Part of the rapid and profound socio-economic change experienced was the development of a Scandinavian type welfare state in Finland. In about 25 years the country developed universal old age, sickness, disability and unemployment benefit systems and started the expansion of a public day care system for children and long term care for the elderly. The existing public education and culture systems were rapidly expanded and a universal health care system was set up. By 1985, it was possible to include Finland in the group of small, prosperous and egalitarian Nordic countries, still somewhat poorer and less generous than the older sisters Sweden and Denmark [31].

### Primary care

In the late 1960's and the early 1970's, the challenge of the Finnish health policy was often articulated by asking: "Why does a country with Europe's healthiest children have the sickest middle-aged male population?" International comparative statistics had indicated that the country was at the European top in terms of low child mortality, but the adult population, particularly males, was dying younger than most other West European adult populations. The positive health status of children was understood as an outcome of a universal, strong and preventive maternal, child and school health system. The health system for the adult population was criticized for being too hospital centered [32,33]. The context of rapid socio-economic change, left-center-coalition government and a perspective of rapid overall development of the welfare state was a fertile growing ground for extending the example of universal, public and preventive child and maternal care to the adult population as well.

The Primary Health Care Act of 1971 started the building of multi-professional and multi-functional local health centers to carry forward the idea of "people's health work" at the local level [34]. It took about 25 years to build health centers throughout the whole country. Thus, in Finland the idea was not restricted to demonstration projects or particular regions as in some other countries, from which the idea of health center was learned [35]. The North Carelia Project, which received widespread international recognition as an example of broad community action for public health initiated by the local health centers [36], was developed as a demonstration project specifically to reduce the high mortality rate from cardio-vascular diseases, in the rural and less prosperous part of Finland.

Given this background, the WHO concept of Primary Health Care as expressed in the Alma-Ata Declaration (1978) was not foreign to Finnish health policy experts. Rather, many of them felt that Finns were pioneers significantly contributing to the development of the WHO policy and demonstrating its applicability also in the Northern hemisphere [21,37].

However, transforming into practice the radical idea of the local health center carrying out "people's health work" was not a simple task. Since the initial expansion phase, the developmental activities and reforms of the health centers have mostly focused on improving the medical cure and care functions [19]. According to some evaluators, health promotion, community-based prevention and public health have largely been pushed to the margins. The emphasis of main reforms addressing the health centers have focused on the management of diseases, division of labour between health centers and hospitals and the development of the GP function in medical care [35]. Thus, the radical concept of Alma-Ata was, in practice, transformed into a normalized concept of primary medical care.

### The community approach

The North Carelia project was and continues to be the best known Finnish example of community action for public health. However, the evaluators of Finnish health promotion policy have repeatedly expressed critical assessment of the leadership and implementation of community action at the local level [19,20]. It has not been mentioned as the strong or innovative part of the Finnish health promotion policy.

Finland also used to be a dissident in resisting the managerialistic idea advocated by the WHO Regional Office for Europe to manage health promotion policy by setting the policy aims in the form of numerical health improvement targets [38].

At the beginning of the 2000s, reference to the role and responsibility of local actors and local community is an essential part of health policy rhetoric [39]. The latest national health promotion programme "Health 2015" [18] is also built around numerical health improvement targets. Thus, we may conclude that both the managerialistic approach and the community approach to the redesign of health policy in the welfare state have been introduced to national health promotion policy rhetoric. However, at the same time as they are present, the evaluators have indicated that these approaches are not effectively implemented.

### Healthy public policy

One aspect of the rapid expansion of the Finnish welfare state in the early 1970s was the idea of improving people's health through a comprehensive planning system of all public sectors. Health indicators were to be used to provide feedback on the health impact of developments in the various public sectors and policies in these sectors should be adjusted accordingly [40]. Alcohol taxation and restrictions on its availability had already been used in Finland, mainly to reduce alcohol related criminality and social problems, but now the same policies were motivated primarily by public health concerns [41]. A comprehensive nutrition policy to change the traditional Finnish diet rich in fatty dairy products and poor in vegetables and fruit was developed. In addition to health education, policies such as shifting the priorities in the subsidies of agricultural products and negotiating changes in the dietary practices of the catering services in the schools and workplaces were used to reduce the consumption of high fat dairy products [42,43]. Tobacco control policies were developed as a flagship of the new health promotion policy applying high excise taxation, restrictions in the availability of tobacco and a ban on advertising. This policy was continuously tightened from the Tobacco Law of 1977 to the late 1990's [44]. Environmental health was also a rapidly developing sector, both as a part of occupational health and as a part of the development of overall environmental legislation and administration, particularly in the 1980s.

The Finnish record on developing policies outside the health sector to promote health has been referred to in placing the country among the forerunners of the Health for All policy in Europe [19,20]. In any case, Finland may be taken as an example of combining ambitious and rapid welfare state building with the ambition of promoting health through the development of the health impact of other policy sectors. It is less obvious that Finland could be taken as an example of how to do this in more mature welfare states. We may, rather, argue that the maturing of the Finnish welfare state from the late 1980's on has been paralleled by growing problems in the development of healthy public policies. The most dramatic example is the dismantling of the traditional Nordic alcohol control policies in the process of redesigning the welfare state under the pressures of European single market legislation and globalization [45]. The existence and at least partly increasing inequity in health between different socio-economic population groups has also been taken as an indication of the less successful development of healthy public policies [46]. Paradoxically, the strengthening of the capacity of the sector to promote health has also separated it from the mainstream health promotion policy. Development of environmental policy and policy administration has contributed to the growing distance in policy discourse and policy communities of environmental and public health. The latest international evaluation of the Finnish national health promotion policy [20] gave a critical assessment of the capacity of health policy makers to assess and influence the policies of other sectors.

### Portugal and the HFA challenge

The Carnation Revolution in 1974 ended a long period of authoritarian rule in Portugal and opened the door to the democratization of the country. As in the other Southern European countries, the democratic Constitution was of a progressive nature while conferring wide economic, social and cultural rights and duties on the citizens [47]. The Constitution that came into force in 1976 aimed at the creation of a welfare state as a political form of transition to a socialist state and society [48]. Although the goal of a socialist, classless society was removed from the Constitution in its reform in 1982, the state's responsibilities to guarantee the economic, social and cultural rights of its citizens were left untouched [49]. Welfare state remained the ultimate goal, but the socialist model was changed to the model of social protection the European Economic Community (EEC) advocated [50,15].

The Southern European welfare state is a relatively recent addition to the conceptual map of European welfare state models. Many southern countries' present day characteristics are related to the legacy of authoritarianism, as well as to the historically strong presence of the Catholic Church [51]. Leibfried sees the weak institutionalization of constitutional promises of social rights as a characteristic feature of Southern European welfare states [47]. The term semi- institutionalized welfare state can be used to describe the whole of the Southern European welfare state that has been built up in principle, yet not implemented in practice. On the other hand it is recognized that southern welfare states have during recent decades been catching up the more developed European welfare systems [47,51]. But in spite of the catching-up effect and the overall pressure towards convergence of social policies in the European Union, Southern European countries seem to maintain a relatively distinct type of welfare state [52,53]. Portuguese welfare state development seems to follow the southern pattern, and Portugal is here analyzed from the viewpoint of the Southern European welfare state type.

The notions of *semi-institutionalization *and *catching up-effect *conceptualize the Southern European welfare state on the one hand as a developing (vs. mature) welfare state and on the other hand as following a different path than the more northern European welfare states [See [47,52]]. The attempts to institutionalize welfare state in Southern Europe occurred simultaneously with the era of welfare state crisis. Consequently, the crisis rhetoric was assumed in Portugal in the initial phases of welfare state development. Thus the welfare state was declared to be in a state of crisis before it actually even existed [53]. Due to the dynamics of *crisis before maturation*, welfare state has remained to some extent a semi- institutionalized promise until the present day.

The development of Portuguese health policy can be broadly divided into two historical phases that are linked to the general welfare state development. The first period from the beginning of the 18^th ^century until 1971 was dominated by preventive public health policies. Through general preventive measures, such as sanitary education, environmental sanitation, hygiene, mental hygiene and sickness prevention "sanitary police" (*polícia sanitária*) aimed at governing the health of the nation. Preventive policies were directed towards the collectivity and they benefited the individual citizen only indirectly. Publicly provided health care services were tied to the clientele of social assistance and were only available to poor people until 1971, when the right to health care was legally defined to be the right of every citizen [54]. The reform bill of Health and Assistance (*Reforma de Saúde e Assistência*) established in 1971 marked the beginning of the second phase of health policies [55]. The consolidation of the universal right to health care in the Constitution and in the National Health Service (NHS) (*Sistema Nacional de Saúde*) law in 1979 [56] signified the strengthening of the social citizenship rights and changed not only the nature of health policies, but also the general nature of the Portuguese welfare state. The qualitative change in the welfare policies from the distributive to productive policies happened precisely in the area of health [53].

### Primary care

Maternal and child health were already part of health policy during the authoritarian era, and women's and children's health was also included into the primary health care concept established with the Reform of Health and Assistance [57]. However these programs were limited to the health education and medical monitoring of women's and children's health during and after pregnancy as family planning was prohibited for political and religious reasons until 1974. A right to family planning was legally defined in the Constitution of 1976 [58]. The integration of family planning into primary health care has widened the scope of maternal and infant health policies in Portugal. Since 1979 Portugal has been collaborating actively with WHO/UNFPA in improving services in family planning [21,59]. In Portuguese health strategy reproductive issues are included in various priority areas. The importance of social policies directed to women, children and family is recognized in the strategy as well as in the government programs. The policies concerned with maternal and child health have developed during the last three decades into policies of reproductive health. The indicators of maternal and child mortality have improved significantly and are on the level of other EU countries [60].

The reform of Health and Assistance aimed at creating a nationwide network of local level health centers that were supposed to provide primary health care services for the entire population [61]. Although the full implementation of this reform was hindered due to political and organizational obstacles, it is seen to mark the beginning of a new era of expansion in Portuguese public health policy [62,63]. This reform included most of the principles of primary health care recognized in the Alma-Ata Declaration seven years later [63,64]. The building of a primary health care network was further consolidated in the Constitution and in NHS law. The process of building up a primary health care network was on the Government's health policy agenda from the beginning of the 1970's until 1985 (15). Analysis of scientific texts and reports on the development of Portuguese public health policy as well as the expert interviews conducted for this study in 2003 indicate that although the Declaration of Alma-Ata was used to legitimise the development of the primary health care system – at least on the level of policy stream – the adaptation of the primary health care-concept presented in Alma-Ata did not change the national policy line.

A right to health care has been an essential part of the democratization process, strengthening social citizenship. Nevertheless the democratization of health care has not been linear; health was politicized following the creation of the public NHS. The critical welfare state philosophy of the liberal political cycle (1985–1995) affected the content of health policies by favoring privatizations of health care during the term of office of the Social Democrats (centre-right party) [63,64]. Due to continuing political and financial problems in the implementation of NHS, difficulties in access to health care services have persisted as a health policy problem. This situation has in its turn kept the development of the health care system and medical care approach in the center of the problem stream feeding the political agenda. According to some of the public health experts interviewed the clinical, curative approach of health care gained more control in the health sector's internal power sharing during the liberal cycle and at the same time the position of public health declined. The analysis of the government programs proves that at the same time the development of primary health care disappeared from governments' agenda. The crisis period of public health policy lasted a decade (1985–1995) [61]. However as the institutionalization of health care has signified the permanent centrality of services on the health policy agenda, not even the crisis period of public health did signified a great break in terms of health promotion in policy documents. Indeed some health education campaigns were launched during the crisis period [15].

The Portuguese Journal of Public Health (*Revista Portuguesa de Saúde Pública*) published a special issue dedicated to HFA in 1988. In the Editorial of the journal it is suggested that HFA2000 should in Portugal have as an objective rather "adequate health care for all" than "health for all" [65]. The general health service orientation of health promotion and disease prevention is also present on the level of government programs. The clinical, treatment-centered ethos typical of the expansion period of the health care system is dominant in the government programs 1976–2002. Health promotion and disease prevention are conceptualized as activities of primary health care and they are seen to be implemented by the medical and nursing professions [15]. Concentration on the primary health care element of the HFA-program is not only a Portuguese specialty; other Southern European countries, such as Spain and Greece, have also put weight on the development of primary health care [59]. The first health strategy is likewise disease-oriented (14 out of 27 of the priority areas are diseases), and since the health service sector is seen as the main actor of health promotion policy, the means are mainly biomedical or educative. Emphasizing rather the individual level than the structural level seems to be a more general Southern European feature in public health policies [66].

### The community approach

The Ottawa Charter calls the countries to strengthen community action. However, it does not explicitly define what is meant by the concept of community. In the social policy literature the term community is often understood to refer either to the network of family members, friends and neighborhoods, or to civil society, understood as a complex of social associations and non-governmental organizations. The archetype of Southern European welfare state carries the connotation of the strong and traditional role of community in welfare provision. [67] However, most comparative studies fail to mention that during the authoritarian era the civil society element of community was repressed, as free associations were prohibited by law. In Portugal only a few religious associations connected to the Catholic Church were approved by the state. Since 1974 the number of associations acting in the field of social and health issues has expanded. [68] Often the call to strengthen community action is seen from the perspective of the welfare state crisis debates. However, in Portugal the growth of the civil society element of community was not an answer to the welfare state crisis as such, but its growth should be located in the context of the recent liberation from state repression. Yet Sousa Santos [69] argues that the state restricts true citizen participation and the functioning of those associations created after 1974 as it continues to support conservative religious organizations.

In the Portuguese health strategy (1999) private institutions of social solidarity (Instituições Particulares da Solidariedade Social) and non-governmental organizations (Organizações Não-governamentais) are recognized as the main representative categories of community. Strengthening partnerships with these organizations is seen as indispensable for achieving the goals set. Although these organizations are also identified as doing health promotion work, they are mainly actors in curing and caring. The second community level actor identified as relevant for health promotion activity is the local level of public administration. Direct citizen participation (e.g. user/consumer/patients' associations) and the need to cooperate with syndicates and health professionals are also mentioned in the strategy. However, they are not given any significant role in the program implementation. All these community categories identifiable in the health strategy seem to match the current categorizations of community actors and their partners in the social sector [See [68]].

The fourth category of community action for health promotion manifest in the form of setting-based projects of Healthy Cities (WHO), the Health Promoting Schools- network (WHO & EU) and Healthy Workplaces (EU). The first Healthy City was established in 1995 and now there are 9 cities belonging to the national network of Healthy Cities [70]. The Health Promoting Schools- network was initiated in 1994 and reaches currently one third of pupils in the public education system [71]. These projects represent the model of community action that is unique for the domain of health promotion. This kind of community based action model targets the whole population of a certain community, while the traditional actors in social and health sectors concentrate on caring for and curing those who are in need of care. Targeted solidarity of traditional community action is challenged by universal equality dominant in these health promotion projects. The model of community action adapted with these projects introduced new ideology and forms of organization into the sphere of public health. When analyzing the adaptation of HFA in a timeframe it seems that the community level adapted HFA philosophy before the national level.

### Healthy Public Policies

The Ottawa Charter emphasizes the role of policy as a factor promoting healthy choices. In other words, this means that health should be taken into consideration in all public policies. When analyzing the Portuguese development in relation to intersectoral policies, there is action in conventional intersectoral issues, such as tobacco, alcohol and nutrition, but it does not seem that any major development has happened in these policy domains. The project of Healthy Schools and the overall health education campaigns are based on interministerial cooperation and pacts between the Ministry of Health and the Ministry of Education. Intersectoral work is also carried out in the field of drug addiction [14,15]. In this section we focus on one case of public policies, that of sanitation, and observe its development in the welfare state development context.

Portuguese public health indicators have shown remarkable improvements during the last three decades. The fact that public health indicators have been improving side by side with general socio- economic indicators has led researchers to conclude that although the creation of NHS and the improved access to health care have influenced the positive evolution of the health status of the Portuguese population, these improvements are greatly connected to general improvements in economic and social conditions, such as education, income and living standard, housing, sanitation, hygiene, and transport infrastructures [72,73,54]. These improvements occurred in the context of the expansion of the welfare state. In this process some of the core issues of the ancient sanitary police, such as matters of basic sanitation, have conceptualized more clearly under respective sectoral policies, out of the national health policy agenda. This reflects the administrative differentiation of state functions and sectoral differentiation of respective policies that typifies the expansion of the welfare state.

Basic sanitation (*saneamento basico*) has been a priority in Portuguese post- authoritarian development policy, however in the government programs (1976–2002) basic sanitation is not recognized as a priority of health policy. Although in some programs environmental conditions and habitation are seen to influence public health and the welfare of the population, basic sanitation is not explicitly considered either as a health policy problem, or as a goal or means. Basic sanitation is not conceptualized as an issue of health policy, it is not explicitly on the government's health policy agenda, neither is health used as an argument to improve it in other sectors. Only in the XIII Government Program (1995–1999) are water quality and the intersectoral action needed to reach it mentioned in the section dealing with health policy. Apart from this, the issue of basic sanitation has become conceptualized as an issue of renovation of infrastructure, and this discourse has constituted it as a policy of infrastructure and renovation. In the Regional Development Plan (2^nd ^Community Support Framework 1994–1999) basic sanitation is conceptualized as an issue of environment and no reference is made to health [74]. In the national health strategy, healthy environments refer to social environments and basic sanitation is not conceptualized as a policy action area. The differentiation of sanitation from the domain of health policy implies that although a change clearly came about in the content of "healthy public policies", it did not happen towards new public health as the improvement of sanitation was not justified by health reasons. Some of the recent documents [75] imply that in recent years the development has begun to turn in a different direction as issues of basic sanitation are again included in the domain of health policies.

## Discussion and conclusions

"Health for All" was developed as an international synthesis of emerging health policy ideas of the 1970's, sometimes conceptualized as "the new public health". Reflecting both the many roots of the concept and the many different contexts to which it was to be adapted, different interpretations of HFA have coexisted. The Alma-Ata Declaration was adapted to combining new public health with local socio-economic development in the developing countries. The HFA targets of the WHO European Region and the Ottawa Charter combine the new public health with the reform demands of state capitalistic and state socialistic welfare states. The target approach is closer to the managerial reform agenda while the Ottawa approach seems to lean more on the community empowerment agenda.

HFA was launched to contribute to the development of national health policies. Thus it may be used as a standard for evaluating national health policies and health promotion policies, as has been done in some studies inspired by the WHO [7-10]. However, understanding HFA as a synthesis of many policy tendencies and allowing different contents for different policy contexts makes such direct comparisons between national policies and WHO documents problematic. In the policy transfer perspective the role of the WHO (or, for that matter, of the EU) may not be that of an international policy leadership but, rather, that of an international policy mediator.

We have tried to trace the impact of HFA on the development of the Finnish and Portuguese health policies. The Finnish development of "people's health work" and local health centers was clearly inspired by the same ideas as the primary health care concept of the Alma-Ata Declaration. The Portuguese health policy ideology expressed in the reforms of 1970's also comprehended the ideas of Alma-Ata Declaration. However, neither of these can be seen as a transfer from WHO to the member states. Rather, the Finns claim that the direction of the transfer was from Finland to WHO. The Portuguese primary care concept also had its own national roots, e.g. in the pre-revolution development of maternal and child health.

The subsequent development of primary health care in both countries indicates that the Alma-Ata idea of broad primary care tends to contradict the welfare state reforms inspired by the ideas of the New Public Management. This context tends to reduce primary health care to primary *medical *care. The impact of this change in the welfare state context may be identified both in Finland and Portugal from the 1980's on as well as in comparing the primary care concepts of the Alma-Ata HFA and the HFA targets of the WHO Europe. At the same time, the aim of the Ottawa Charter of reorienting health services towards health promotion does not seem to have guided primary care development in either country.

Thus the development of primary care in both countries has been in dialogue with the HFA. However, what primary care means in the framework of HFA has changed over time and the dialogue cannot be simplified into the unidirectional transfer of HFA policy from WHO to member states.

Dialogue or interaction are also appropriate concepts to describe the relationship between WHO and the two countries with regard to developing a community approach in health promotion policy. First of all, the different variants of HFA locate "community" in different contexts. In Alma-Ata, community is the totality of local actors without making distinctions between economic, social and health actors or private and public actors. The European HFA target documents [4,6] see community as a partner or a cluster of partners to the health sector and public authorities. The Ottawa Charter seems to be build around the idea of community empowerment and increasingly participative health policy making. The Finnish community approach as expressed in the North Carelia project, in the cooperation of the public health sector with the traditional public health associations and in the emphasis on local public sector action, seems to be quite close to the approach of the European HFA targets. Both the broad community concept of Alma-Ata and the community empowerment approach of Ottawa seem more alien to Finnish health policy strategies.

A number of welfare state characterizations [e.g. [47,67]] create expectations that we should find, in Portugal, a strong role of traditional communities strongly linked to the Catholic Church in health promotion policy. Such an expectation may fail to recognize the historical legacy of the authoritarian Salazar regime, which, while keeping close linkage to the Catholic Church, was quite a state centered regime that did not allow strong independent community action. Our analysis indicates that the role of community action in health promotion is not particularly eminent in Portugal, either in governmental health policy documents [14,15] or according to the opinion of public health experts [61,76]. The activity of the Catholic Church and religiously inspired organizations in health promotion is, however, visible [77,78]. But so is also the attempt of the government to conceptualize community action through projects such as Healthy Cities and Health Promoting Schools, where community action is led or arranged by the public authorities.

Thus, whatever is meant by the community approach in health promotion policy, Finland and Portugal do not seem to be strong examples of policy development following the initiative of HFA. We could not identify policy transfer other than in participation in the Healthy Cities and other "health settings" projects.

Healthy Public Policy was our third focus in health promotion policy. The concept was raised in the European HFA document in 1985. In Alma-Ata the integration of health and other policies is extended much further and no specific concept resembling health public policy is needed.

The Finnish health promotion strategy has included a number of public policies outside the health sector, particularly with regard to alcohol, tobacco, nutrition and physical exercise. We could not identify any specific impact of the different HFAs of the WHO on these policies. Rather, there seems to be growing pressure to restrict the use of the impact of other sectors in alcohol control. At the same time, the distance between the mainstream health promotion policy and environmental policy seems to be growing, although the public health impact of environmental policies is obvious. Thus, with the exception of tobacco policy, the idea of healthy public policy may even experience increasing problems, although this is not so far reflected in the development of the health status of the population.

The rapid positive development of the health status of the Portuguese population during the last 30 years reflects the rapid improvement of the sanitary conditions as well as of the social determinants of health [72,73,54]. Sanitary policy, including both preventive services such as vaccinations and health education, as well as improvement of environmental and housing conditions, has been the most significant aspect of Portuguese healthy public policy. However, the analysis of health policy documents indicates that Portugal has also experienced a distancing of environmental policies and health policies, that is: a trend antagonistic to the ideas of the different version of HFA. Other public policies, including tobacco and alcohol control and nutrition policies are weakly developed in Portugal. Thus, we cannot identify any significant transfer mediated by the WHO in Portugal either.

At the beginning of 1970's public health indicators showed that Finland and Portugal were lagging behind the majority of Western European countries in terms of public health indicators. Both defined this distance from the Western European level as the core health policy problem [15]. This way of defining the policy problem has clearly contributed to the fact that both countries have looked to international organizations and international comparison for their policy development.

The Finnish health policy expert community has often referred to WHO and Finland has been an active member of the European region of this organization. In the 1980's, it even took the responsibility for acting as a pilot country for the national development of HFA in Europe [37]. Thus there has been much interaction between WHO and Finland in health policy development. Our analysis indicates that this interaction cannot be understood as policy transfer and that it has influenced Finnish health policy development much less than is often assumed.

For the Portuguese government documents, the EU and the idea of a "European welfare state" has been the reference much more often that the WHO [15]. However, Portugal has also been in dialogue with the WHO in health policy development, although not to the same extent as Finland.

We have also asked what conditions the adoption of HFA policy in the two countries. Our analysis indicates that the phase of welfare state development matters a lot. The ambitious welfare state development period in the late 1960's and the 1970's in Finland was a good basis for adopting the ambitious idea of "people's health work" and setting far-reaching aims for the development of the health impact of all public policies. Much of the Finnish health promotion policy development until the 1990's is rooted in the initiatives of this period. HFA, as expressed in the Alma-Ata Declaration and in the later versions of HFA were taken in Finland as international evidence in support of the policy choices already made in the country. Portugal also had courageous ambitions of developing a European welfare state, after the Carnation Revolution and the call of Alma-Ata was heard in this context. While Finland was fairly successful in building a universalistic institutional welfare state of the Scandinavian type, Portugal seems to have so far ended up in what Leibfried (1992) calls a semi-institutional welfare state. This may be a good explanation for the continuity of health promotion policy in Finland, in contrast to the discontinuity in Portugal which also is reflected in the concept "semi-institutional".

Both HFA and the two countries examined have also been influenced by the end of the "Golden Age of the Welfare State" [79]. The differences between the Alma-Ata approach and those of the Ottawa Charter and European HFA expressed in policy targets is not only the difference between global and Europe or OECD. It is also a difference between the ambitions of the Golden Age and the post-expansion period [80] of the Welfare State. Now the political agenda is dominated by the idea of reforming the (existing) welfare state. We have linked the Ottawa approach to a reform agenda emphasizing community empowerment and the European HFA targets approach to the more managerialistic reform agenda. While we can identify the impact of the managerialistic agenda in both countries to the reduction of "primary health care" to "primary medical care", we are more hesitant regarding the impact of the community empowerment agenda on the health policy development in the two countries.

The development of health promotion policy in the two countries has also been related to changes in politics, particularly to changes in the political composition and orientation of the national governments. In this regard, the Portuguese development has been stormier with a radical regime shift in the Carnation Revolution, starting a new regime inspired by socialist visions, followed by a turn to liberal conservative governments ten years later. The Socialist Party's victory in the elections of 1995 after ten years in opposition signified a change in social policy orientation once again [50,81]. However, there does not seem to be any significant changes in the health promotion policy even if the government has published health strategy. The Finnish political development has been much less stormy. The tradition of coalition governments which normally include both the Social Democratic Party and some parties of the bourgeois side has strengthened continuity rather than radical turns in Finnish health policy. However, within this continuity, an incremental movement from welfare state expansion to post-expansive welfare state reform policy may be identified [82-84].

Our analysis does not give a clear picture of the significance of politics in the adoption of HFA in the two countries. We may assume that the continuity in the Finnish politics has contributed to the continuity in the Finland-WHO dialogue and interaction as expressed, e.g., in the reviews of WHO-teams of Finnish health policy development [19,20]. The more stormy political development in Portugal may also have caused more discontinuity in the WHO relationship. However, the level of interaction was not a direct indicator of significant policy transfer. Rather, our analysis shows that the political context and its changes in countries probably impacts on which version of HFA is adopted. Thus, during the dominance of politics in support of more radical or expansionist welfare state development, Alma-Ata seems to have been the preferred version of HFA, while the European HFA target approach may be more feasible in the post-expansive welfare state politics.

In Kingdonian (1995) terms, we may sum up that the health policy problem of both Finland and Portugal, being European laggards in the 1970's, caused them to be open to transfer of policies from abroad. Thus, the "problem stream" was ready for policy transfer. The "policy stream" seems also to have been ready for a certain kind of transfer, but only for those versions or elements of HFA that could be fitted into the specific policy contexts of the countries. HFA as such was not a dynamo of policy change in either country. The "politics stream" changed in both countries so that the window for radical policy changes was closed fairly soon. After that, if any political window was open, it was only for incremental changes in line with the post-expansive welfare state reform agenda.

## Competing interests

This study is a part of a research project called "Finnish National Health Promotion Policy from an International Comparative Perspective", which has been financed by the Academy of Finland.

JL acted as a scientist in WHO Centre for Health Policy, Brussels, in 1999.

## Authors' contributions

JL is responsible for the analysis of Finnish policy while LTG has done the analysis on Portugal and drafted the other parts of the article.
